# Dystocia in cats under UK primary emergency veterinary care: epidemiology, clinical management and outcomes

**DOI:** 10.1111/jsap.70120

**Published:** 2026-04-24

**Authors:** L. Leicester, D. G. O’Neill

**Affiliations:** ^1^ Vets Now Ltd. Dunfermline UK; ^2^ Pathobiology and Population Sciences The Royal Veterinary College Hatfield UK

## Abstract

**Objectives:**

To explore the epidemiology, clinical management and outcomes for feline dystocia cases presented to UK primary emergency veterinary care.

**Materials and Methods:**

Retrospective observational cross‐sectional study. Demographic and clinical data were extracted on feline dystocia cases under emergency care (2017 to 2023). Risk factor analysis used multivariable binary logistic regression.

**Results:**

Among 118,168 queens, 1102 dystocia cases were identified, giving an overall incidence risk of 0.93% (95% CI 0.88 to 0.99). Purebred queens had 2.53 (95% CI 2.16 to 2.97) times odds of dystocia compared to crossbred queens. Brachycephalic queens had 3.27 (95% CI 2.68 to 3.98) times odds of dystocia compared to non‐brachycephalic queens. Individual breeds with the highest odds of dystocia compared with crossbred queens were Devon Rex (OR: 10.38, 95% CI 4.61 to 23.37), Tonkinese (OR: 8.27, 95% CI 2.92 to 23.43), Birman (OR: 7.40, 95% CI 3.17 to 17.28), Exotic (OR: 6.29, 95% CI 2.23 to 17.73) and British Short Hair (OR: 3.81, 95% CI 2.96 to 4.89). Initial medical dystocia management included oxytocin in 386/1102 (35.02%) cases, with 104/386 (26.94%) of oxytocin‐treated cases progressing to caesarean section. Overall, caesarean section was performed in 394/1102 (35.75%) queens. Litters of 5 to 6 kittens showed 6.54 (4.36 to 9.81) times odds of caesarean section compared with litters of 1 to 2. During emergency care, 38 of 1102 (3.45%) dystocic queens died. The neonatal mortality rate was 1072 of 2784 (38.51%) kittens born across all dystocia cases.

**Clinical Significance:**

These findings indicate substantial impact from dystocia on feline maternal and neonatal welfare and survival. Awareness of higher dystocia risk in purebred, brachycephalic and certain breed queens can help veterinary staff and breeders better predict and prepare for maternal and neonatal care requirements.

## INTRODUCTION

Dystocia is defined as difficulty with normal vaginal delivery of a neonate from the uterus (Davidson, 2010) and is a common reproductive emergency in cats (Černá et al., 2024; Gunn‐Moore & Thrusfield, 1995; Holst et al., 2017), with potential to cause high‐maternal and neonatal morbidity and mortality (Bailin et al., 2022; Holst et al., 2017; Smith, 2012). As well as representing a substantial feline welfare risk, dystocia can also be distressing for owners and breeders (Gunn‐Moore & Thrusfield, 1995).

Incidence of feline dystocia in breeding cat populations has been reported to range between 5.8% to 14.9%, based on survey data from multiple countries (Černá et al., 2024; Gunn‐Moore & Thrusfield, 1995; Holst & Frössling, 2009; Sparkes et al., 2006). Feline dystocia represented 2.3% of reimbursed insurance claims in queens in a Swedish study (Holst et al., 2017). However, despite being a medical and/or surgical condition that often requires emergency veterinary care (Bailin et al., 2022), there is limited information on the incidence of feline dystocia among the subset of cats presenting for emergency veterinary care in the UK.

Purebred status (Gunn‐Moore & Thrusfield, 1995; Holst et al., 2017) and brachycephaly (Gunn‐Moore & Thrusfield, 1995) are reported risk factors for dystocia in cats. However, previous exploration of individual breed risk has yielded somewhat conflicting results. A UK breeding population survey reported higher dystocia odds in Persian, Siamese‐type and Devon Rex cats compared to other breeds (Gunn‐Moore & Thrusfield, 1995). In contrast, a study based on reimbursed pet insurance claims in Sweden identified British Shorthair, Oriental group, Ragdoll, Birman and Abyssinian breeds at higher dystocia risk than other purebreds (Holst et al., 2017). However, some other studies using survey data from breeding cats in various countries failed to identify significant breed‐related risk of dystocia (Černá et al., 2024; Holst & Frössling, 2009; Sparkes et al., 2006). Given these conflicting findings, and that some of this information is now quite dated, there is a need for more recent evidence on breed‐specific risk of feline dystocia to help breeders and owners better understand relevant breed‐related welfare risks.

Maternal proportional mortality rates varying between 2.0% and 5.7% of queens experiencing dystocia have been reported (Bailin et al., 2022; Holst et al., 2017; Sendag et al., 2025). Neonatal mortality rates from 16.0% to 25.0% of kittens born to overall births have been reported in different feline breeding populations (Černá et al., 2024; Fournier et al., 2017; Romagnoli et al., 2019; Sparkes et al., 2006), with 34.0% mortality reported among kittens born to dystocic cats presenting for emergency care (Bailin et al., 2022). High maternal and kitten mortality associated with feline dystocia highlights the value of a stronger evidence base on frequency, risk factors and clinical management strategies, to improve outcomes and mitigate the welfare impacts.

Non‐surgical clinical interventions for feline dystocia include manual assistance of delivery that can be supported by medical management using synthetic oxytocin and/or calcium gluconate to augment uterine contractions (Gendler et al., 2007; Pretzer, 2008; Wiebe & Howard, 2009). The proportion of medically managed dystocic cats that progress to surgical intervention was reported as 70.1% of 155 dystocic cats presenting to a Swedish veterinary hospital (Ekstrand & Linde‐Forsberg, 1994), and 28.6% of 35 dystocic cats at the Michigan State University emergency service, USA (Bailin et al., 2022). Parallel canine and feline dystocia studies at a Swedish veterinary hospital reported higher failure rates for medical management of dystocia in cats than in dogs, with initial medical management progressing to surgical management in 70.1% of dystocic cats (Ekstrand & Linde‐Forsberg, 1994) compared to 62.4% of dystocic dogs (Darvelid & Linde‐Forsberg, 1994). In the UK, a study of 701 canine dystocia cases presenting between 2012 and 2014 to the same emergency‐care provider as the current study reported that 32.6% of bitches managed medically with oxytocin progressed to surgical management (O'Neill et al., 2019), although a parallel study on medically managed dystocic queens has remained lacking until now.

Proportional caesarean section rates are reported to vary across different female breeding cat populations. Of 1056 litters documented by UK pedigree cat breeders of 14 different breeds in a questionnaire survey, 8.0% of litters were reported born by caesarean section (Sparkes et al., 2006). A UK breeding population survey of 2928 litters from 735 pedigree and non‐pedigree queens reported that 74% of 169 dystocic litters were born surgically (Gunn‐Moore & Thrusfield, 1995). In an owner and breeder health survey on over 8000 Finnish purebred and non‐purebred cats, caesarean section prevalence among non‐purebred cats was reported as 0.13%, but was significantly higher at 7% among purebred cats, and higher still in Birmans, Burmese and Siamese‐type queens (Vapalahti et al., 2016). Among dystocic cats presenting for veterinary care in different countries, proportional caesarean section has been reported from 37.1% to 79.4% (Bailin et al., 2022; Ekstrand & Linde‐Forsberg, 1994; Holst et al., 2017; Sendag et al., 2025), but generalising these data to UK cat populations is problematic due to varying geographic norms and views on the ethics of routine neutering of companion animals. There is little information on clinical management outcomes for dystocic cats in veterinary caseloads in the UK, although this information would support veterinarians in benchmarking and evidence‐based clinical decision‐making for feline dystocia cases (RCVS Knowledge, 2024).

Clinical data routinely recorded on practice management systems on emergency veterinary care populations can provide a valuable research resource for studies of dystocia (Leicester et al., 2023; O'Neill et al., 2017, 2019), which often presents as a medical and/or surgical emergency (Bailin et al., 2022; Holst et al., 2017; Smith, 2012). Findings from research on dystocia cases in a primary emergency‐care caseload could generalise well to dystocia cases in the wider population of animals presenting for non‐emergency primary care in the UK, with some acknowledged limitations, (O'Neill et al., 2017, 2019), given that the emergency‐care cats represent a subset of the wider primary‐care population.

The current study aimed to explore the clinical records of cats under veterinary care at Vets Now from 1 January 2017 to 31 December 2023 to investigate the epidemiology, clinical management and outcomes of dystocia in cats. The specific objectives were to determine the incidence risk of feline dystocia; evaluate risk factors for feline dystocia and caesarean section; and determine clinical management and outcomes from dystocia for queens and kittens. It was hypothesised that medical management with oxytocin progresses to surgical management more commonly in feline dystocia cases under primary emergency veterinary care than the 32.6% previously reported in dogs (O'Neill et al., 2019).

## MATERIALS AND METHODS

Vets Now delivers primary out‐of‐hours emergency care at over 60 sites for companion animals registered at more than 1700 primary‐care partner practices around the UK, and annually treats over 200,000 companion animals (Vets Now, 2025). Information on clinical care is documented electronically using a bespoke practice manage system (PMS) called Helix.

### Study design

A retrospective observational cross‐sectional study was designed to estimate the incidence risk and risk factors for feline dystocia using data extracted from Helix electronic health records (EHRs). The study population included all cats recorded as female with at least one EHR on Helix, which had attended Vets Now from 1 January 2017 to 31 December 2023. Ethical approval was granted by Nottingham Trent University (reference number ID1805073). Sample size calculations estimated that a cross‐sectional study would require a sample size of 18,759 queens to report a 2% incidence risk with a 0.2% confidence limit (Dean et al., 2013), assuming a UK cat population size of 5,500,000 queens (PDSA, 2024a) and design effect of 1.0 (Kirkwood & Sterne, 2003).

The case definition for feline dystocia was a queen presenting for emergency veterinary clinical care related to parturition, with at least part of one fetus recorded by the attending clinician as retained internally at initial presentation (Leicester et al., 2023; O'Neill et al., 2017, 2019).

The Helix database was interrogated using a Structured Query Language (SQL) query utilising Microsoft SQL Server Management Studio (Microsoft Corporation, 2024a) to identify all queens with Helix EHRs, which attended for emergency care from 1 January 2017 to 31 December 2023. Dystocia case‐finding involved a 2‐step process. Firstly, to identify candidate feline dystocia cases, an iterative development process was used to validate terms related to dystocia that were used to search four Helix data fields: client‐reported “presenting complaint”; clinician‐reported “presenting complaint”; clinician‐reported “provisional diagnosis” that used VeNom Coding standardised terminology (VeNom Coding Group, 2024); and clinician‐reported drugs, procedures and free‐text clinical notes in the “clinical journal” (Table 1).

**Table 1:** Search terms used to search four practice management system data fields to identify candidate feline dystocia cases

Helix data field	Validated search terms
Client‐reported “presenting complaint”	Trouble giving birth
Clinician‐reported “provisional diagnosis”	Dystocia
Clinician‐reported drug products, tests, procedures	Kittening assistance, caesarean, oxytocin
Clinician‐reported free‐text clinical notes	Apgar, breach, breech, caesarean, caesarian, cesarean, cesarian, contraction, distocia, dystocia, fetal, foetal, kittening assistance, maternal, neonatal, neonate, oxytocin, parturition, placenta, still birth, still born, stillbirth, stillborn, trouble giving birth

Secondly, candidate dystocia cases were randomly ordered using the NEWID function in SQL (Microsoft Corporation, 2024a). Clinical notes and uploaded documents relating to clinical care were manually reviewed in Helix to evaluate each candidate case against the feline dystocia case definition and to extract details on clinical care and outcomes for confirmed cases. Candidate cases not meeting the case definition for feline dystocia were returned to the non‐case set. Ethical approval was granted by Nottingham Trent University (reference number ID1805073).

### Data extracted

All available information on breed, date of birth, date of the clinical care event and clinic attended were extracted for all study cats. The breed information was mapped to a VetCompass standardised cat breed list that further categorised breed into breed‐derived variables: purebred status and brachycephalic status (VetCompass, 2025). The “purebred” category included cat breeds (Lipinski et al., 2008) recognised by Governing Council of the Cat Fancy (2024), Cat Fancier's Association (2024), Felis Britannica (2025) or The International Cat Association (2025). The “crossbred” breed category included cats recorded as “crossbreed”, “Domestic Short Hair”, “Domestic Medium Hair” and “Domestic Long Hair”. Cats with missing breed data were categorised as “no breed recorded”. Individual breed names with five or more dystocia cases were retained in the analysis as individual breeds along with a grouping of crossbred cats, while all remaining named breeds were grouped as “other”. Cats recorded as American Short Hair, Bombay, British Long Hair, British Short Hair, Burmese, Chinchilla, Devon Rex, Exotic, Himalayan, Persian and Scottish Fold were categorised as brachycephalic (Cat Fanciers' Association, 2024; Felis Britannica, 2025; Governing Council of the Cat Fancy, 2024; The International Cat Association, 2025).

Age was calculated at the date of the first clinical care event for the episode of dystocia for dystocia cases. For non‐dystocia cases with one clinical care event, age was calculated at the date of that clinical care event, and for non‐dystocia cases with more than one clinical care event, age was calculated at the midpoint between the dates of the first and last clinical care event (O'Neill et al., 2017). Age (years) for all queens was categorised into four groups: “0 to 2.9”, “3.0 to 5.9”, “≥6.0” and “no age recorded”.

Bodyweight data were extracted for the purpose of estimating doses of oxytocin administered in IU/kg, but were not included in risk analysis because the recorded bodyweight of dystocic queens often also included variable numbers of intrauterine kittens.

Clinical care data extraction consistency was optimised through the development of a structured list of clinical care questions that aimed to capture key aspects of the clinical management and outcomes of feline dystocia cases (Table 2). These questions focused on medical and surgical interventions, maternal and neonatal survival and treatment decisions during emergency care. The list was refined during a pilot study of 40 dystocia cases to support clarity, relevance and applicability across varied clinical contexts. Each question was designed to be answerable from the EHR and to reflect meaningful clinical events. For example, oxytocin (Oxytocin‐S, 10 IU/mL; MSD Animal Health) administration was recorded only when used to assist fetal passage, excluding post‐partum use. Caesarean sections were classified as elective or emergency, and neonatal outcomes were categorised as stillborn, liveborn or liveborn but dying prior to discharge. Maternal mortality included both euthanasia and unassisted death, with recorded reasons for euthanasia extracted where applicable. Definitions for each question are provided in Table 2.

**Table 2:** Clinical care questions used to extract data from electronic health records of 1102 feline dystocia cases presenting to Vets Now for emergency care from 1 January 2017 to 31 December 2023

Clinical questions	Definitions
Was oxytocin administered?	Oxytocin (10 IU/mL) to assist with the passage of a fetus from the queen, but not post‐partum for uterine involution or passage of placenta
Oxytocin first quantity administered (IU/cat)?	
Oxytocin first dose administered (IU/kg)?	
Was a caesarean section performed?	Laparotomy to extract at least one fetus from the queen ± ovariohysterectomy
Was the caesarean section elective?	
During the dystocia episode, how many kittens were stillborn, liveborn or liveborn and died before discharge from emergency care?	Stillborn: born deceased before or during the episode of emergency care
	Liveborn: born alive before or during the episode of emergency care (evidence of voluntary respiration with or without resuscitation)
	Liveborn and died: born alive before or during the episode of emergency care, but did not survive to discharge
Did the queen die during the dystocia episode?	Includes dying by euthanasia and dying unassisted during treatment
If euthanased, what was the predominant reason for euthanasia?	

### Statistical analysis

Data were checked and cleaned in Excel (Microsoft Corporation, 2024b), and statistical analyses were conducted using Epitools Epidemiological Calculators (Epitools, 2024) and IBM SPSS Statistics Version 29.0 (IBM Corporation, 2023). Demographic descriptive statistics were reported separately for dystocia cases and dystocia non‐cases for breed, purebred status, brachycephalic status and age. The mean and standard deviation [SD] were reported for continuous variables with normal distribution and the median, interquartile range [IQR] and range for non‐normally distributed data. Normality was assessed by visualisation of histograms. The Mann–Whitney *U* test was used for univariable age comparison between cases and non‐cases. Proportional caesarean rates among dystocic cats of different breeds were compared using the chi‐square test (IBM Corporation, 2023). Kitten mortality was compared across age categories and breed using one‐way ANOVA (IBM Corporation, 2023). For hypothesis testing, the probability of feline dystocia cases in the current study being medically managed with oxytocin and progressing to surgical management was compared to previously reported 32.6% for 701 canine dystocia cases presenting for emergency care at Vets Now between 2012 and 2014 (O'Neill et al., 2019) using the chi‐square test (IBM Corporation, 2023).

Risk factor analysis for dystocia as an outcome used multivariable binary logistic regression modelling in SPSS (IBM Corporation, 2023). Demographic risk factors (breed, purebred status, brachycephalic status, age) with liberal association (*P* < .200) with dystocia in univariable analysis were considered in multivariable modelling. Model development used Wald automated backward stepwise elimination. Since breed was a factor of primary interest, purebred and brachycephalic status, which were breed‐derived and therefore highly correlated with breed, were initially excluded from multivariable modelling but were explored iteratively by replacing breed in the final breed‐focused model (O'Neill et al., 2024). Clinic was evaluated as a random effect (O'Neill et al., 2017, 2019). Pairwise interaction effects were evaluated for the variables in the final breed‐focused model. The Hosmer–Lemeshow test was used to evaluate the model fit (Archer et al., 2007). Statistical significance was set at *P* < .05.

Risk factor analysis for an outcome of caesarean section was conducted within the subset of queens with dystocia. Univariable binary logistic regression was used to evaluate associations for demographic (breed, age) and clinical (litter size, initial oxytocin dose) variables. Only litter size met the liberal inclusion threshold (*P* < .200), and therefore, no multivariable model was developed.

## RESULTS

### Dystocia incidence

There were 1102 dystocia cases identified from a study population of 118,168 queens that attended for emergency care between 1 January 2017 and 31 December 2023, yielding a dystocia incidence risk among all queens of 0.93% (95% CI 0.88 to 0.99).

Breed data were available for 58,588/118,168 (49.6%) queens overall, and age data were available for 112,236/118,168 (95.0%) queens overall. Table 3 reports descriptive results for age and breed. The median age of cats in the study was 7.01 years (IQR 2.00 to 13.01, range 0.003 to 27.02). Median age (years) differed statistically significantly between dystocia cases (median 1.92, IQR 1.00 to 3.00, range 0.41 to 12.73) and non‐cases (median 7.01, IQR 2.00 to 13.01, range 0.003 to 27.02) (Mann–Whitney *U* test *z* = −28.985, *P* < .001).

**Table 3:** Demographic descriptive and univariable binary logistic regression modelling results (with 95% confidence interval [*CI]) for 118,168 queens presenting to Vets Now for emergency care from 1 January 2017 to 31 December 2023 that included 1102 dystocia cases and 117,066 dystocia non‐cases

Variable	Category	All study cats no. (%)	Cases no. (%)	Non‐cases no. (%)	Odds ratio	95% CI*	Category *P*‐value	Variable *P*‐value
Breed	Crossbred	47,486 (40.2)	362 (32.8)	47,124 (40.3)	Base			**<.001**
	British Short Hair	2311 (2.0)	79 (7.2)	2232 (1.9)	4.61	3.60 to 5.90	**<.001**	
	Other	1680 (1.4)	28 (2.5)	1652 (1.4)	2.41	1.50 to 3.25	**<.001**	
	Ragdoll	1576 (1.3)	28 (2.5)	1548 (1.3)	2.35	1.60 to 3.47	**<.001**	
	Benga l	1389 (1.2)	28 (2.5)	1361 (1.2)	2.68	1.82 to 3.95	**<.001**	
	Persian	1000 (0.8)	25 (2.3)	975 (0.8)	3.33	2.22 to 5.03	**<.001**	
	Maine Coon	906 (0.8)	15 (1.4)	891 (0.8)	2.19	1.30 to 3.69	.**003**	
	Siamese	897 (0.8)	21 (1.9)	876 (0.7)	3.11	2.00 to 4.87	**<.001**	
	Burmese	500 (0.4)	8 (0.7)	492 (0.4)	2.12	1.05 to 4.29	.**037**	
	Sphynx	265 (0.2)	8 (0.7)	257 (0.2)	4.04	1.99 to 8.25	**<.001**	
	Birman	202 (0.2)	6 (0.5)	196 (0.2)	3.99	1.76 to 9.04	**<.001**	
	Tonkinese	109 (0.1)	4 (0.4)	105 (0.1)	1.82	1.82 to 13.53	.**002**	
	Scottish Fold	96 (0.1)	5 (0.5)	91 (0.1)	7.14	2.89 to 17.70	**<.001**	
	Devon Rex	88 (0.1)	7 (0.6)	81 (0.1)	11.25	5.16 to 24.52	**<.001**	
	Exotic	80 (0.1)	4 (0.4)	76 (0.1)	6.85	2.49 to 18.83	**<.001**	
	No breed recorded	59,583 (50.4)	474 (43.0)	59,109 (50.5)	1.04	0.91 to 1.20	.540	
Purebred status	Crossbred	47,486 (40.2)	362 (32.8)	47,124 (40.3)	Base			**<.001**
	Purebred	11,099 (9.4)	266 (24.1)	10,833 (9.3)	3.20	2.73 to 3.75	**<.001**	
	No breed recorded	59,583 (50.4)	474 (43.0)	59,109 (50.5)	1.04	0.91 to 1.20	.540	
Brachycephalic status	Non‐brachycephalic	54,273 (45.9)	495 (44.9)	53,778 (45.9)	Base			
	Brachycephalic	4312 (3.6)	133 (12.1)	4179 (3.6)	3.46	2.85 to 4.20		
	No breed recorded	59,583 (50.4)	474 (43.0)	59,109 (50.5)	0.87	0.77 to 0.99		
Age (years)	3.0 to 5.9	16,422 (13.9)	214 (19.4)	16,208 (13.8)	Base			
	0.0 to 2.9	32,616 (27.6)	757 (68.7)	31,859 (27.2)	1.80	1.54 to 2.10		
	≥6.0	63,195 (53.5)	46 (4.2)	63,149 (53.9)	0.06	0.04 to 0.08		
	No age recorded	5935 (5.0)	85 (7.7)	5850 (5.0)	1.10	0.85 to 1.42	.437	

*P*‐values < .05 are presented in bold

The most common breeds among the 1102 dystocia cases were crossbred (*n* = 362, 32.8%), British Shorthair (*n* = 79, 7.2%), Ragdoll (*n* = 28, 2.5%) and Bengal (*n* = 28, 2.5%). The most common breeds among the 117,066 non‐cases were crossbred (*n* = 47,124, 40.3%), British Shorthair (*n* = 2232, 1.9%), Ragdoll (*n* = 1548, 1.3%) and Bengal (*n* = 1361, 1.2%) (Table 3).

**FIG 1:**
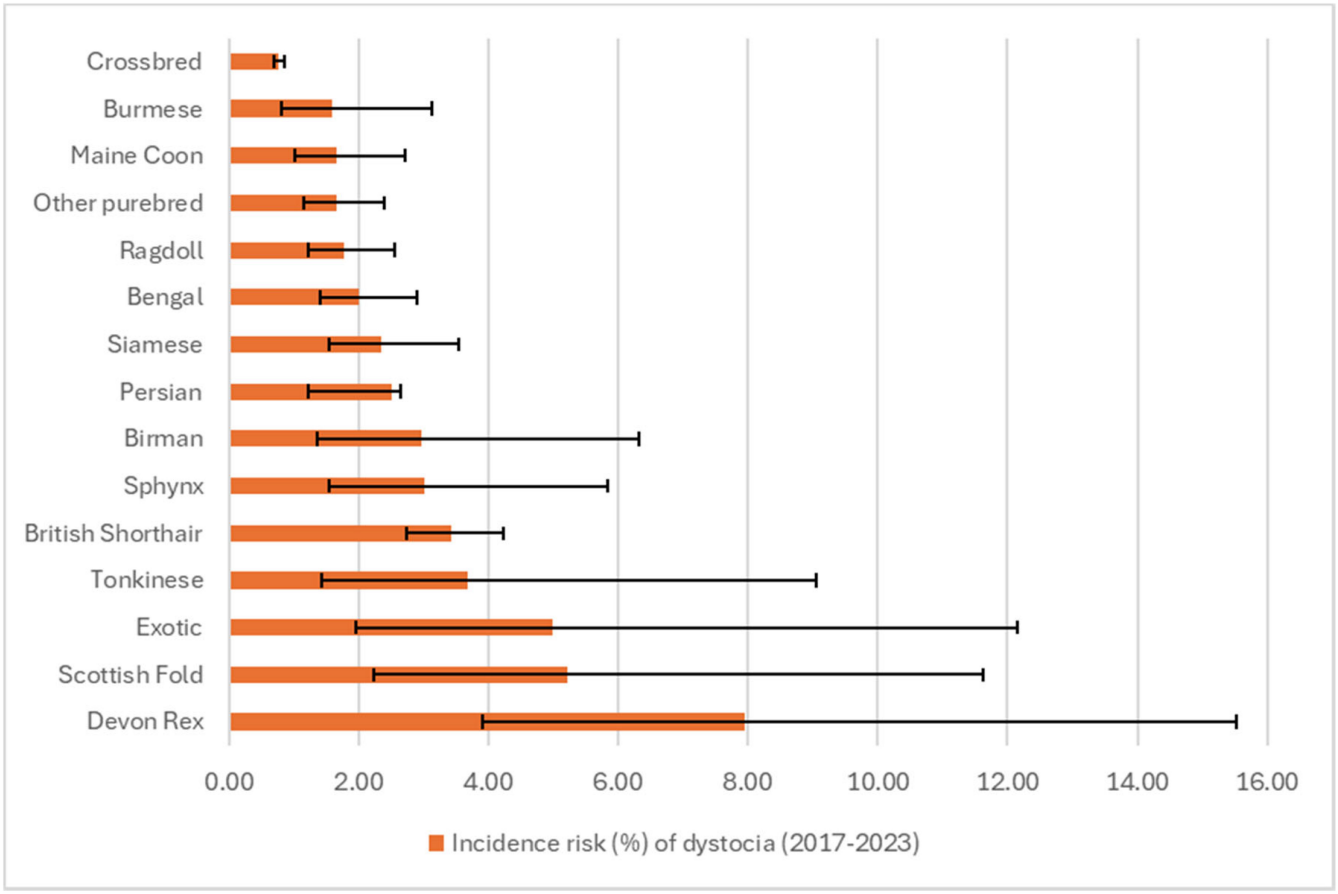
Incidence risk (% with 95% confidence interval) of dystocia by breed among 118,168 queens presenting to Vets Now for emergency care from 1 January 2017 to 31 December 2023. Orange bar represents incidence risk, and horizontal black line represents 95% confidence interval.

Breeds with the highest incidence risk were Devon Rex (7.95%, 95% CI 3.91 to 15.52), Scottish Fold (5.21%, 95% CI 2.24 to 11.62) and Exotic (5.00, 95% CI 1.96 to 12.16) (*P* < .001). Incidence risk among crossbred cats was 0.76% (95% CI 0.69 to 0.85) (Fig 1).

### Risk factors for dystocia

Univariable logistic regression modelling identified four demographic variables with liberally significant (*P* < .200) association with dystocia: breed, purebred status, brachycephalic status and age (Table 3). The final breed‐focused multivariable model for dystocia included two variables: breed and age. Inclusion of clinic as a random effect did not improve the model fit, and therefore, clinic was not included in the final model. No significant interactions were identified in the final model. The final model showed no evidence of poor model fit (Hosmer–Lemeshow test statistic: *P* = .161). After accounting for the effects of age, breeds with the highest odds for dystocia were Devon Rex (OR: 10.38, 95% CI 4.61 to 23.37), Tonkinese (OR: 8.27, 95% CI 2.92 to 23.43) and Birman (OR: 7.40, 95% CI 3.17 to 17.28). Queens aged 0.0 to 2.9 years had 1.79 (95% CI 1.53 to 2.09) the odds of presenting with dystocia compared with queens aged 3.0 to 5.9, and dystocia odds decreased in cats aged six years or older (Table 4).

**Table 4:** Final breed‐focused multivariable logistic regression results (% with 95% confidence interval [*CI]) for risk factors associated with dystocia among 118,168 queens presenting to Vets Now for emergency care from 1 January 2017 to 31 December 2023

Variable	Category	Odds ratio	95% CI*	*P*‐value
Breed	Crossbred	Base		
	Devon Rex	10.38	4.61 to 23.37	**<.001**
	Tonkinese	8.27	2.92 to 23.43	**<.001**
	Birman	7.40	3.17 to 17.28	**<.001**
	Exotic	6.29	2.23 to 17.73	**<.001**
	British Short Hair	3.81	2.96 to 4.89	**<.001**
	Persian	3.60	2.37 to 5.45	**<.001**
	Scottish Fold	3.28	1.32 to 8.12	.**010**
	Siamese	3.22	2.05 to 5.05	**<.001**
	Burmese	2.88	1.41 to 5.89	.**004**
	Sphynx	2.23	1.09 to 4.56	.**028**
	Bengal	1.95	1.32 to 2.89	**<.001**
	Other	1.74	1.18 to 2.57	.**006**
	Maine Coon	1.46	0.86 to 2.46	.159
	Ragdoll	1.41	0.96 to 2.09	.082
	No breed recorded	1.04	0.91 to 1.20	.545
Age (years)	3.0 to 5.9	Base		
	0.0 to 2.9	1.79	1.53 to 2.09	**<.001**
	≥6.0	0.06	0.04 to 0.08	**<.001**
	No age recorded	1.16	0.90 to 1.49	.260

*P*‐values < .05 are presented in bold

As explained in the methods, breed was iteratively replaced by purebred and brachycephalic status in the final breed‐focused model. After replacing breed, purebred queens had 2.53 (95% CI 2.16 to 2.97) times the odds of dystocia compared with crossbred queens, and brachycephalic queens had 3.27 (95% CI 2.68 to 3.98) times the odds of dystocia compared with non‐brachycephalic queens (Table 5).

**Table 5:** Multivariable logistic regression results (with 95% confidence interval [*CI]) for risk factors that replaced breed in the final breed‐focused model for association with dystocia among 118,168 queens presenting to Vets Now for emergency care from 1 January 2017 to 31 December 2023

Variable	Category	Odds ratio	95% CI*	*P*‐value
Purebred status	Crossbred	Base		
	Purebred	2.53	2.16 to 2.97	**<.001**
	No breed recorded	1.04	0.91 to 1.20	.543
Brachycephalic status	Non‐brachycephalic	Base		
	Brachycephalic	3.27	2.68 to 3.98	**<.001**
	No breed recorded	0.91	0.80 to 1.03	.148

*P*‐values < .05 are presented in bold

### Clinical management

Medical management of dystocia with oxytocin was attempted in 386/1102 (35.0%) cases, of which 233/386 (60.4%) had oxytocin dosage information (IU/kg) available. The median oxytocin dose (IU/kg) at first administration was 0.40 IU/kg (IQR 0.25 to 0.59, range 0.03 to 2.00). Of those 386 dystocia cases administered oxytocin, 104/386 (26.9%) progressed to surgical management.

Caesarean section was performed in 394/1102 (35.8%) dystocic queens overall, with 268/394 (72.6%) of the completed caesareans including an ovariohysterectomy procedure. None of the caesarean sections were recorded as elective procedures. The proportional caesarean section rates among dystocic cats varied across the breeds (*P* < .001). Breeds with the highest proportional caesarean section rates included Siamese (61.90%, 95% CI 40.88 to 79.25), Bengal (53.57%, 95% CI 35.81 to 70.47), Birman (50.00%, 95% CI 18.76 to 81.24), Burmese (50.00%, 95% CI 21.52 to 78.48) and Ragdoll (50.00%, 95% CI 32.63 to 67.37) (Table 6). Discharge from emergency care occurred prior to completion of parturition in 474/1102 (43.0%) feline dystocia cases.

**Table 6:** Proportional caesarean section rates by breed (with 95% confidence interval [*CI]) among 1102 feline dystocia cases presenting to Vets Now for emergency care from 1 January 2017 to 31 December 2023

Breed	No. dystocia cases	No. of dystocia with caesarean section	Proportional caesarean section rate (%)	95% CI*
Siamese	21	13	61.90	40.88 to 79.25
Bengal	28	15	53.57	35.81 to 70.47
Birman	6	3	50.00	18.76 to 81.24
Burmese	8	4	50.00	21.52 to 78.48
Ragdoll	28	14	50.00	32.63 to 67.37
Tonkinese	4	2	50.00	05.00 to 85.00
British Shorthair	79	35	44.30	33.87 to 55.27
Persian	25	11	44.00	33.87 to 55.27
Devon Rex	7	3	42.86	15.82 to 74.95
Crossbred	362	151	41.71	36.75 to 46.85
Scottish Fold	5	2	40.00	11.76 to 76.93
Maine Coon	15	5	33.33	15.18 to 58.29
Other	28	8	28.57	18.55 to 47.98
Exotic	4	1	25.00	4.56 to 69.94
Sphynx	8	2	25.00	7.15 to 59.07
No breed recorded	474	125	26.37	22.61 to 30.52

### Risk factors for caesarean section

As described in the methods, litter size was the only variable significantly associated with an outcome of undergoing caesarean section among the dystocia cases, so univariable results only are reported (Table 7). Dystocic litters of 5 to 6 kittens showed 6.54 (4.36 to 9.81) times the odds of caesarean section compared with litters of 1 to 2 kittens.

**Table 7:** Univariable logistic regression modelling results (with 95% confidence interval [*CI]) for risk factor association with caesarean section among 1102 feline dystocia cases presenting to Vets Now for emergency care from 1 January 2017 to 31 December 2023

Variable	Category	Dystocia cases no.	Dystocia with caesarean section no. (%)	Odds ratio	95% CI*	Category *P*‐value
Breed	Crossbred	362	151 (41.7)	Base		
	British Shorthair	79	35 (44.3)	1.11	0.68 to 1.82	.673
	Ragdoll	28	14 (50)	1.40	0.65 to 3.02	.394
	Other	28	8 (28.6)	0.56	0.24 to 1.30	.178
	Bengal	28	15 (53.6)	1.61	0.75 to 3.49	.225
	Persian	25	11 (44)	1.10	0.49 to 2.49	.823
	Siamese	21	13 (61.9)	2.27	0.92 to 5.61	.076
	Maine Coon	15	5 (33.3)	0.70	0.23 to 2.09	.520
	Sphynx	8	2 (25)	0.47	0.09 to 2.34	.353
	Burmese	8	4 (50)	1.40	0.34 to 5.68	.640
	Devon Rex	7	3 (42.9)	1.05	0.23 to 4.75	.952
	Birman	6	3 (50)	1.40	0.28 to 7.02	.685
	Scottish Fold	5	2 (40)	0.93	0.15 to 5.64	.939
	Tonkinese	4	2 (50)	1.40	0.20 to 10.03	.739
	Exotic	4	1 (25)	0.47	0.05 to 4.52	.510
	No breed recorded	474	125 (26.4)	0.50	0.37 to 0.67	**<.001**
Purebred status	Crossbred	362	151 (41.7)	Base		
	Purebred	266	118 (44.4)	1.11	0.81 to 1.53	.508
	No breed recorded	474	125 (26.4)	0.50	0.37 to 0.67	**<.001**
Brachycephalic status	Non‐brachycephalic	495	211 (42.6)	Base		
	Brachycephalic	133	58 (43.6)	1.04	0.71 to 1.53	.839
	No breed recorded	474	125 (26.4)	0.48	0.37 to 0.63	**<.001**
Litter size	1 to 2	345	75 (21.7)	Base		
	3 to 4	324	159 (49.1)	3.47	2.48 to 4.85	**<.001**
	5 to 6	169	109 (64.5)	6.54	4.36 to 9.81	**<.001**
	≥7	35	21 (60.0)	5.40	2.62 to 11.13	**<.001**
	Litter size not recorded	106	30 (28.3)	1.42	0.87 to 2.33	.163
Oxytocin first dose IU/kg	0 to 0.19	35	16 (45.7)	Base		
	0.2 to 0.39	80	29 (36.3)	0.68	0.30 to 1.51	.340
	0.4 to 0.59	61	21 (34.4)	0.62	0.27 to 1.46	.276
	0.6 to 0.79	19	8 (42.1)	0.86	0.28 to 2.67	.799
	0.8 to 0.99	14	5 (35.7)	0.66	0.18 to 2.37	.524
	≥1.0	24	14 (58.3)	1.66	0.58 to 4.75	.342
Age (years)	3.0 to 5.9	214	86 (40.2)	Base		
	0.0 to 2.9	757	265 (35.0)	0.80	0.59 to 1.10	.164
	≥6.0	46	15 (32.6)	0.72	0.37 to 1.41	.340
	No age recorded	85	28 (32.9)	0.73	0.43 to 1.24	.245

*P*‐values < .05 are presented in bold

### Maternal mortality

Overall, 38 of 1102 (3.5%) queens with dystocia died before discharge. Of these, 36/38 (94.7%) were euthanased and 2/38 (5.3%) died unassisted. Poor medical prognoses were recorded as the predominant rationale for the euthanasia decision in 30/36 (83.3%) euthanased queens, while financial constraints for the pet owner to support ongoing treatment were recorded as the predominant rationale for the euthanasia decision in 6/36 (16.7%) euthanased queens.

### Kitten mortality

Outcome information for kittens born during the episode of dystocia, up to discharge from emergency care, were available in 996/1102 (90.4%) dystocia cases. Mortality information on kittens born at home to queens which later received care for dystocia was included. Overall kitten mortality up to discharge from emergency care was 1072/2784 (38.5%) kittens born. Table 8 reports overall and litter‐based kitten outcomes, and Table 9 reports kitten outcomes by breed, queen age and litter size.

**Table 8:** Descriptive statistics for birth and survival to discharge outcomes of 2784 kittens born to 996 feline dystocia cases presenting to Vets Now for emergency care from 1 January 2017 to 31 December 2023 (*Column %)

Kitten outcome	Total no. kittens	No. kittens per litter		
	Kittens no. (%)*	Median	Interquartile range	Range
Stillborn	874 (31.4)	1	0 to 1	0 to 6
Liveborn	1910 (68.6)	2	0 to 3	0 to 9
Liveborn and died	198 (10.4)	0	0 to 0	0 to 5
Died before discharge	1072 (38.5)	1	0 to 2	0 to 10
Survived to discharge	1712 (61.5)	1	0 to 3	0 to 9
Total born	2784 (100.0)	3	1 to 4	0 to 11

**Table 9:** Descriptive statistics for birth and survival to discharge outcomes of 2784 kittens born to 996 feline dystocia cases presenting to Vets Now for emergency care from 1 January 2017 to 31 December 2023, by breed, queen age and litter size

Variable	Category	Feline dystocia cases no.	Dystocia cases with caesarean section no. (%)	Median kittens per litter no.	Total kittens born no.	Stillborn kittens no. (%)	Liveborn kittens which died no. (%)	Total kitten mortality no. (%)
Breed	Crossbred	329	137 (41.6)	3	900	274 (30.4)	67 (7.4)	341 (37.9)
	British Short Hair	74	34 (45.9)	3	237	72 (30.4)	23 (9.7)	95 (40.1)
	Other purebred	28	8 (28.6)	4	68	22 (32.4)	2 (2.9)	24 (35.3)
	Bengal	26	14 (53.8)	3	80	27 (33.8)	9 (11.3)	36 (45.0)
	Ragdoll	24	11 (45.8)	3	72	18 (25.0)	2 (2.8)	20 (27.8)
	Persian	22	10 (45.5)	2	49	18 (36.7)	4 (8.2)	22 (44.9)
	Siamese	18	10 (55.6)	3	68	17 (25.0)	4 (5.9)	11 (30.6)
	Maine Coon	13	5 (38.5)	3	54	13 (24.1)	3 (5.6)	16 (29.6)
	Burmese	8	4 (50.0)	3.5	36	8 (22.2)	3 (8.3)	21 (30.9)
	Devon Rex	7	3 (42.9)	2	15	2 (13.3)	2 (13.3)	4 (26.7)
	Birman	6	3 (50.0)	4	20	6 (30.0)	1 (5.0)	7 (35.0)
	Sphynx	6	2 (33.3)	1	8	2 (25.0)	0 (0.0)	2 (25.0)
	Tonkinese	4	2 (50)	1	14	5 (35.7)	0 (0.0)	5 (35.7)
	Exotic	4	1 (25)	3	11	3 (27.3)	0 (0.0)	3 (27.3)
	Scottish Fold	3	2 (66.7)	4	13	6 (46.2)	0 (0.0)	6 (46.2)
	No breed recorded	425	118 (27.8)	3	1139	381 (33.5)	78 (6.8)	459 (40.3)
Age (years)	0.0 to 2.9	684	246 (36.0)	3	1897	579 (30.5)	133 (7.0)	712 (37.53)
	3.0 to 5.9	194	79 (40.7)	3	553	183 (33.1)	48 (8.7)	231 (41.77)
	≥6.0	44	14 (31.8)	3	114	41 (36.0)	8 (7.0)	49 (42.98)
	No age recorded	74	25 (33.8)	3	220	71 (32.3)	9 (4.1)	80 (36.36)
Litter size at discharge	0	123						
	1	199	21 (10.6)		199	96 (48.2)	14 (7.0)	110 (55.3)
	2	146	54 (37.4)		292	111 (38.0)	13 (4.5)	124 (42.5)
	3	185	82 (44.3)		555	183 (33.0)	41 (7.4)	224 (40.4)
	4	139	77 (55.4)		556	182 (32.7)	34 (6.1)	216 (38.8)
	5	105	62 (59.0)		525	134 (25.5)	42 (8.0)	176 (33.5)
	6	64	47 (73.4)		384	96 (25.0)	34 (8.9)	130 (33.9)
	7	22	12 (54.5)		154	37 (24.0)	8 (5.2)	45 (29.2)
	8	4	3 (75.0)		32	6 (18.8)	1 (3.1)	7 (21.9)
	9	5	3 (60.0)		45	18 (40.0)	5 (11.1)	23 (51.1)
	10	2	1 (50.0)		20	3 (15.0)	1 (5.0)	4 (20.0)
	11	2	2 (100.0)		22	8 (36.4)	5 (22.7)	13 (59.1)
Total		996	364 (36.5)		2784	874 (31.4)	198 (7.1)	1072 (38.5)

Among the overall 1102 dystocic queens presented for emergency care, no significant difference was detected in kitten mortality rates between age groups (*P* = .891), individual breeds (*P* = .467), purebred and crossbred queens (*P* = .667) or brachycephalic and non‐brachycephalic queens (*P* = .436). Among the subset of 394 dystocic queens managed surgically, no significant differences were detected in kitten mortality rates between age groups, (*P* = .490), individual breeds (*P* = .704), brachycephalic and non‐brachycephalic queens (*P* = .479) or purebred and crossbred queens (*P* = .358).

## DISCUSSION

This study identified feline dystocia as an important contributor to the overall feline emergency caseload, affecting 0.93% of all queens presented for emergency care. Young age, breed, purebred and brachycephalic status are highlighted as risk factors for feline dystocia. Large litter size is shown as a risk factor for caesarean section in dystocic queens. These findings can contribute to the evidence base on feline dystocia, helping owners and clinicians anticipate which cats are more likely to develop dystocia and improve the timeliness of veterinary care for dystocic queens.

The 0.93% incidence risk of feline dystocia reported in the current study is lower than 2.3% reported previously in insured dystocic queens presenting for veterinary care in Sweden (Holst et al., 2017). This disparity highlights the importance of considering the characteristics of the denominator study population when interpreting frequency statistics. The Swedish figure was based on insured queens, while the current study reflects queens presenting for emergency veterinary care in the UK: these are two populations that may differ in risk profiles and healthcare‐seeking behaviours. Despite these differences, both studies likely substantially underreported the true incidence of feline dystocia in the subset of queens at actual risk (*i.e*. those queens who are both entire and pregnant). This is because both studies included neutered queens, which cannot become pregnant, because reliable information on neuter status was unavailable for either study (Holst et al., 2017). Neutered cats account for an estimated 85% of the UK cat population (Cats Protection, 2024a). If this pattern of neuter status holds true for queens presenting for emergency care, only 15% of queens would be at actual risk, suggesting that the true incidence of dystocia among entire queens in the current study could be as high as 6.22%. These findings advocate for enhanced focus on feline reproductive emergencies in both undergraduate and postgraduate veterinary curricula.

Dystocia risk varied widely across common breeds in the current study, with Devon Rex, Tonkinese, Birman, Exotic, British Shorthair, Persian, Scottish Fold and Siamese queens having at least three times the odds of dystocia compared to crossbred queens. However, it should also be noted that the odds ratios for several breeds carried wide confidence intervals, indicating relative underpowering due to low numbers for those breeds. Comparing findings between studies on breed‐related risk of dystocia can be challenging due to differing study populations and case definitions. For example, a Swedish study exploring dystocia risk in insured queens was underpowered for Persians and excluded Sphynx and Bengal cats because these cat breeds appear uncommon in Sweden (Holst et al., 2017), but were relatively common breeds in the current study in the UK. Additionally, selective breeding may progressively alter breed characteristics over time, which could influence the reported breed risk of dystocia in studies carried out at different time points (Černá et al., 2024). However, there is recurring strong evidence for relatively high dystocia risk for four cat breeds also identified in the current study: Birman (Gunn‐Moore & Thrusfield, 1995; Holst et al., 2017), British Shorthair (Holst et al., 2017), Devon Rex (Gunn‐Moore & Thrusfield, 1995) and Siamese (Gunn‐Moore & Thrusfield, 1995; Holst et al., 2017). These findings add to the evidence base of breed‐related health issues in certain purebred cats and suggest that a more robust approach is needed to promote responsible feline breeding practices.

After accounting for the other factors assessed, purebred queens in the current study showed 2.52 times higher odds of dystocia than crossbred queens. This purebred cat predisposition aligns with similar findings from two previous studies (Gunn‐Moore & Thrusfield, 1995; Holst et al., 2017). Breed‐related factors reported to influence dystocia risk include genetic predisposition, and breed‐specific conformational traits such as head shape and pelvic diameter (Monteiro et al., 2013). A study series exploring dystocia among feline breeding cat populations has reported a progressive increase in dystocia incidence in purebred cats since 1995 that could reflect increasing genetic and conformational divergence between purebred cats and their more innately healthy crossbred counterparts kept as pets (Černá et al., 2024; Gunn‐Moore & Thrusfield, 1995; Sparkes et al., 2006). This trend is particularly concerning given the reported increasing ownership of purebred cats in the UK (Cats Protection, 2024a; Roberts et al., 2025).

Brachycephalic queens, which include several popular UK breeds, showed 3.27 times the odds of dystocia compared with non‐brachycephalic queens in the current study, aligning with a 1995 UK study reporting brachycephalic queens with 3.17 times the odds of dystocia compared with mesocephalic queens (Gunn‐Moore & Thrusfield, 1995). These findings add to existing evidence that extreme cranial conformation may have severe health and welfare implications. They could inform breeder education and owner counselling, and support the need for continued attention to breeding practices and public awareness regarding the health impacts associated with extreme conformations (British Veterinary Association, 2026; Federation of European Companion Animal Veterinary Associations, 2018; Roberts et al., 2025; LAGECDogs, 2026).

Scottish Fold queens had the second highest breed incidence of dystocia in the current study (5.2%) and showed 3.28 times the odds of presenting for dystocia compared to crossbred queens. In addition to this dystocia predisposition, Scottish Folds are genetically predisposed to osteochondrodysplasia, which is associated with arthritis and painful progressive limb deformities (Malik et al., 1999; Roberts et al., 2025; Takanosu et al., 2008; Universities Federation for Animal Welfare, 2011; Velie et al., 2023). Despite these health problems, Scottish Folds are reportedly growing in popularity in the UK, with an estimated 3% of all cats acquired between March 2023 and March 2024 reported as being Scottish Folds (Cats Protection, 2024b). Due to their well evidenced health issues, the breeding of Scottish Fold cats is not supported by major UK veterinary and animal welfare organisations (Cats Protection, 2024c; Governing Council of Cat Fancy, 2024; RSPCA, 2024; Universities Federation for Animal Welfare, 2011) and has effectively been banned in Scotland by licensing regulations that prohibit the breeding of cats with certain inherited disorders (Scottish Government, 2021). However, there are currently no equivalent licensing regulations for cat breeding in the rest of the UK (Animal Welfare Committee, 2024; Legislation.gov.uk, 1951). More comprehensive licensing and stronger enforcement of welfare standards for cat breeding could promote responsible cat breeding, especially among cat breeds with multiple health issues. Evidence from the current study strengthens the case for such legislative change.

The current results did not support the study hypothesis that initial clinical management with oxytocin progresses to surgical management more commonly in dystocia cases in cats than the 32.6% previously reported in dogs (O'Neill et al., 2019). Instead, a statistically significant difference in the opposite direction was detected, with only 26.9% of dystocic cats initially managed with oxytocin progressing to surgery. Comparison between the two studies is complicated because 43% of the dystocic cats were discharged from emergency care before parturition was complete, whereas only 27.1% of the dystocia bitches were discharged before parturition was complete (O'Neill et al., 2019). Some of these discharged patients may have subsequently required surgical management, which could have narrowed the gap in proportional surgical management. Additionally, although both studies explored data from the same emergency‐care practice, clinical approaches to dystocia in feline and canine patients may differ due to species differences in available evidence, clinician behaviours and owner preferences. Clinical outcomes in dystocic cats in the current study broadly aligned with a smaller USA study of 35 dystocia cats presenting to a university emergency service, where 29% of cats with initial non‐surgical management progressed to surgery (Bailin et al., 2022), and 42% of dystocic cats were discharged before parturition was completed. Given the financial costs and medical risks (Goethem, 2016) associated with caesarean section, it is not unexpected that non‐surgical management of feline dystocia is a common initial approach in UK primary emergency care. However, with a substantial proportion of dystocic queens being discharged home or to another veterinary care provider before completion of parturition, there is a need for studies that collect follow‐up data on outcomes for this group of queens. More accurate evidence on progression from non‐surgical to surgical management could inform care frameworks and checklists to support clinical decision‐making in feline dystocia cases, reduce morbidity and mortality associated with delays in necessary surgical interventions and assist with identification of dystocia cases where initial non‐surgical management is appropriate.

The breeds with highest proportional risk of caesarean section among the dystocic queens in the current study were Siamese (13/21, 61.9%), Bengal (15/28, 53.6%), Birman (3/6, 50%), Burmese (4/8, 50%) and Ragdoll (14/28, 50%). However, despite some variation in absolute proportional caesarean risk across the breeds, breed was not statistically significant as a risk factor for caesarean section among the cats with dystocia. An absence of evidence for a statistically significant breed effect for caesarean section aligns with other studies that also failed to identify breed with a significant effect on the odds of caesarean section (Černá et al., 2024; Sparkes et al., 2006). Challenges to identifying breed‐related effects in the current study included the 43% missing breed data and severe underpowering of the breed‐based analysis from small sample sizes for several breeds. It is possible that breed truly has no or just a minor influence on the probability of caesarean section and that perhaps other factors such as litter size (Černá et al., 2024; Sparkes et al., 2006), comorbidities (Traas, 2008) and timing of presentation for veterinary care for dystocia (Traas, 2008) may carry much higher effects.

Litter size was the only factor tested that showed a predictive effect on the risk of caesarean section among dystocic queens in the current study. Litters of 5 to 6 had the highest odds, being 6.54 times more likely to have a caesarean section than litters of 1 to 2. This finding contrasts with two earlier studies of purebred breeding cats, which reported queens with smaller litters at higher risk of caesarean section (Černá et al., 2024; Sparkes et al., 2006). A potential explanation for these different results is that the current study population also contained crossbred cats, which may have been less likely to represent intentional breeding (Cats Protection, 2024a). Intentionally bred queens may be more closely monitored during parturition than those with unplanned pregnancies, potentially leading to earlier presentation for veterinary care and caesarean section. This may reflect the higher commercial value of purebred kittens or greater owner involvement in the breeding process. This may have contributed to different patterns of caesarean section occurrence in the current study compared to the studies of purebred populations.

Maternal mortality among dystocic queens was reported at 3.5% in the current study, which is midway between the 2.0% reported in 670 dystocic queens in a study of insured queens in Sweden (Holst et al., 2017), and 5.7% reported in a study of 35 dystocic cats presenting to a USA emergency‐care service (Bailin et al., 2022). The Swedish study only reported on events with a reimbursed insurance claim and did not report on unclaimed events or uninsured queens, so could have underreported maternal mortality (Holst et al., 2017). Either way, with around one in thirty dystocic queens dying as a result of their dystocia, these findings highlight the welfare importance of timely veterinary intervention for feline dystocia (Bailin et al., 2022; Holst et al., 2017; Smith, 2012) and also the value of avoiding pregnancy in queens with predictable increased risk of dystocia. In the current study, 2/38 (5.3%) queens died unassisted during treatment, and 30/38 (79.0%) were euthanased due to perceived poor prognosis. Clinical signs of disease observed in dystocic cats could represent comorbidities, complications of dystocia itself (Holst, 2022) or a mixture of both. In any case, some of these queens that died may have benefitted from receiving veterinary care much earlier during their dystocia episodes. A wider awareness of the risk of maternal mortality in dystocic cats could help promote the importance of timely veterinary care, which could improve feline welfare and potentially reduce the risk of fatal outcomes in dystocic cats. Including detailed information on reported maternal morbidity and mortality for feline dystocia in information resources for cat owners (Cats Protection, 2024b; International Cat Care, 2024; PDSA, 2024b) may help to encourage neutering and prevent unplanned feline pregnancies.

The 38.5% overall kitten mortality, including stillbirths and deaths of liveborn kittens before discharge from care, was concerningly high in the current study. However, this is consistent with the 34% kitten mortality reported in 35 dystocic cats at a Michigan university emergency service, USA, (Bailin et al., 2022). High‐neonatal mortality among dystocic queens in veterinary caseloads highlights the potential value of timely veterinary intervention. While many neonatal losses occur due to stillbirth, there may be opportunities to support early neonatal survival through care practices such as the assessment of newborn kitten vitality through Apgar scoring, to identify at‐risk neonates which require immediate intervention (Axelsson, 2019; Hibaru et al., 2022).

Kitten mortality did not differ significantly between breeds within the 1102 dystocic queens presented for emergency care in the current study. Previous studies of breeding cat populations have reported considerable breed‐related variation in kitten mortality (Černá et al., 2024; Fournier et al., 2017; Romagnoli et al., 2019). Potential explanations for the differences in findings between studies could reflect that the current study included only queens presented for emergency care, and therefore, selected for a population of neonates at high risk of mortality in the first place. Other non‐breed factors that were not included in the current study such as the length of gestation, comorbidities and timing of presentation for veterinary care during the dystocic episode could also have overshadowed any breed‐related effects on kitten mortality. Comparisons between studies is further complicated because the current study included data on kitten mortality only up to discharge from care, whereas the studies of breeding cat populations included kitten mortality up to 6 to 12 weeks (Černá et al., 2024; Fournier et al., 2017; Romagnoli et al., 2019). Further studies, with more accurate methods of breed capture, and which follow outcomes after discharge from care, are needed to understand kitten mortality in primary‐care populations.

There were some limitations to the current study. While the study benefited from access to clinical data with a wide geographical distribution and a large number of dedicated emergency‐care practices, the true population of UK cats that become pregnant and therefore are at risk of dystocia is unknown. The study relied on secondary use of clinical data originally recorded to document details of emergency veterinary care but that was not originally created for research purposes (O'Neill et al., 2014). Breed data were missing in 43% feline dystocia cases. The study was underpowered for some breeds due to low‐breed numbers, so breed‐specific findings should be viewed in this light. Variation in the timing of bodyweight measurements relative to the delivery of kittens may have influenced the accuracy of oxytocin dose estimations (IU/kg), due to the inclusion of varying numbers of intrauterine kittens in some queens at the time of weighing. Protocols for medical management of dystocia were not standardised between sites and were based on individual clinician discretion within a wider contextualised care framework (Skipper et al., 2024). The decision to progress from medical to surgical management of dystocia can be influenced by a variety of factors such perceived prognosis or financial value of the kittens, introducing variability that could affect interpretation of treatment outcomes. The data available for the study did not consistently capture information on whether queens had experienced prior dystocia or undergone previous caesarean sections. These factors have been reported as risk factors for dystocia recurrence in both dogs (Schrank et al., 2022) and cats (Axnér et al., 2025) and may influence later clinical decision‐making and outcomes, particularly regarding the choice of medical *versus* surgical management. Future studies incorporating reproductive history could help clarify its role in dystocia risk and treatment planning. Information on the underlying causes of dystocia were not consistently recorded in the available clinical data, so the proportion of cases involving obstructive dystocia – a contraindication for oxytocin use (NOAH Compendium, 2025) – could not be determined. While this limits interpretation of oxytocin administration, the reported progression from medical to surgical management in this emergency‐care setting hopefully still offers a useful reference point and benchmarking data for clinical decision‐making in other primary‐care settings.

In conclusion, this study reports feline dystocia as a relatively common presentation for emergency veterinary care with some strong demographic risk factors. The results highlight high levels of maternal and neonatal mortality, supporting more focused teaching on feline dystocia within both pre‐ and postgraduate veterinary education. The findings may help support better informed decision‐making among cat owners considering breeding, by raising awareness of the potential risks associated with feline dystocia.

### Author contributions

Data curation: L. Leicester; Conceptualisation: L. Leicester, D. G. O’Neill; Data extraction: L. Leicester; Methods: L. Leicester, D. G. O’Neill; Formal analysis: L. Leicester, D. G. O’Neill; Writing – original draft: L. Leicester; Writing – review and editing: L. Leicester, D. G. O’Neill.

### Conflict of interest

LL is an employee of Vets Now.

### Funding

This study was designed as part of the BSAVA Masters program and was supported by Vets Now.

## Data Availability

The data that support the findings of this study are available from the corresponding author upon reasonable request.
